# Supplementary material for: Sexual Dimorphism in Extracellular Matrix Composition and Viscoelasticity of the Healthy and Inflamed Mouse Brain
Source: Biology (Basel). 2022 Jan 31;11(2):230. doi: 10.3390/biology11020230 (PMC8869215; doi:10.3390/biology11020230)
Supplement: Supplementary file 1 [file biology-11-00230-s001.zip › biology-1537695-supplementary.pdf]

biology MDPI

Supplementary Materials

Table S1: Gene symbols and corresponding primers for TaqMan® assays.

gene symbol	gene name	amplicon length	TaqMan® Assay ID
Lama4	laminin, alpha 4	81	Mm01193660_m1
Lama5	laminin, alpha 5	64	Mm01222029_m1
Col1a1	collagen, type I, alpha 1	89	Mm00801666_g1
Col4a1	collagen, type IV, alpha 1	74	Mm01210125_m1
Fn1	fibronectin 1	58	Mm01256744_m1
Hprt1	hypoxanthine guanine phosphoribosyl transferase	131	Mm03024075_m1

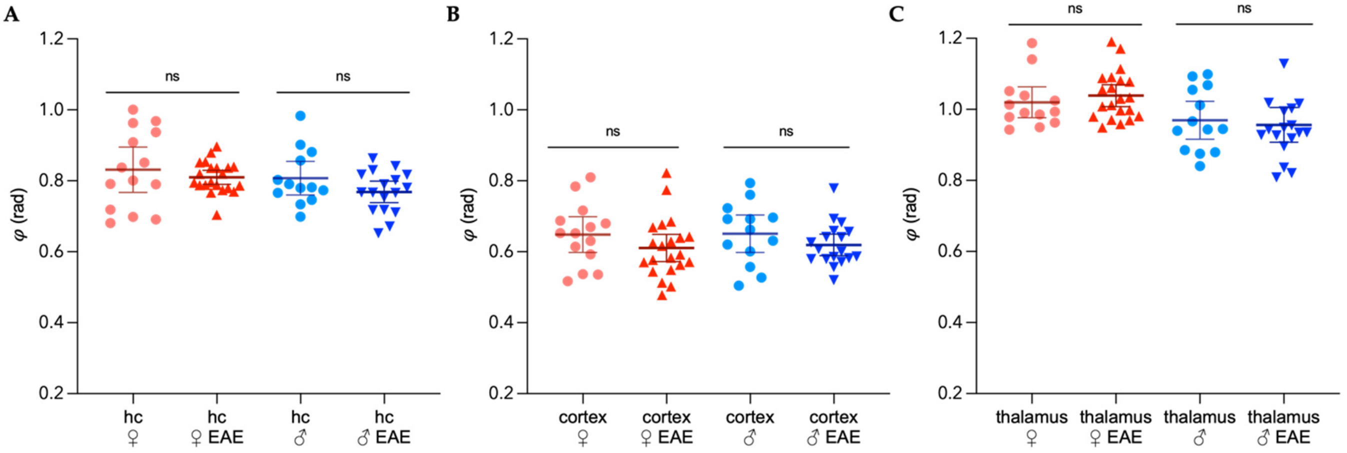

Figure S1. Regional distribution of sex-specific fluidity φ in the midbrain of the mouse in healthy and EAE. (**A**) hippocampus (hc), (**B**) cortex and (**C**) thalamic area; light red = healthy female, dark red = EAE female, light blue = healthy male, dark blue = EAE male. Representation of individual data points with mean and 95%CI. $n_{female\ naive}$ = 14, $n_{male\ naive}$ = 13, $n_{female\ EAE}$ = 21, $n_{male\ EAE}$ = 18. Group comparison performed with unpaired *t* test with Welch correction.

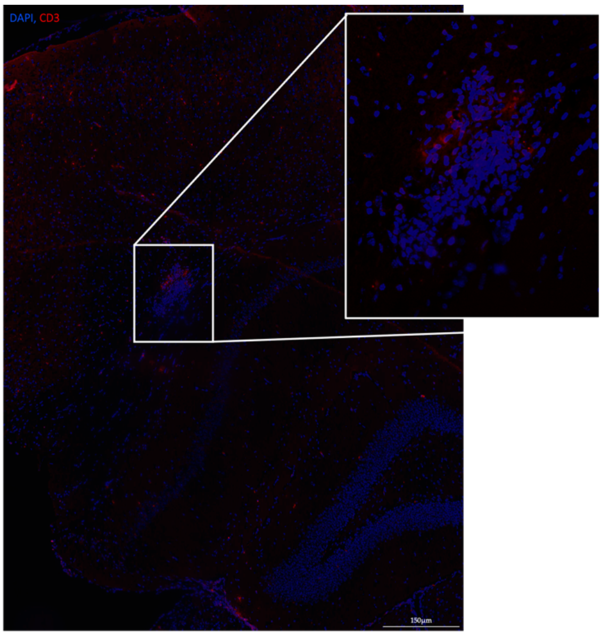

Figure S2. Singular cortical lesion in the midbrain in male EAE, adjacent to the hippocampus. CD3 (red) and DAPI (blue). Scale bar = 150µm.
